# Supplementary material for: Analysis of PPARγ Signaling Activity in Psoriasis
Source: Int J Mol Sci. 2021 Aug 10;22(16):8603. doi: 10.3390/ijms22168603 (PMC8395241; doi:10.3390/ijms22168603)
Supplement: Supplementary file 1 [file ijms-22-08603-s001.zip › Supplemental materials_Analysis of PPARg signaling activity in psoriasis/Pathway models/Models images and html files/PPARG expressed in cells assotiated with psoriasis/225654.html]

fibroblast


# CellType fibroblast

|  |  |
| --- | --- |
| URN | urn:agi-ncimcelltype:C1550637 |
| Total Entities | 71 |
| Connectivity | 13870 |
| Name | fibroblast |

---

|  |  |
| --- | --- |
| ChildConcepts | fibroblast function |
|  | fibroblast adhesion |
|  | fibroblast infiltration |
|  | fibroblast proliferation |
|  | fibroblast migration |
|  | fibroblast apoptosis |
|  | fibrocyte |
|  | cancer-associated fibroblast |
|  | heart fibroblast |
|  | lung fibroblast |
|  | fibroblast differentiation |
|  | fibroblast survival |
|  | fibroblast development |
|  | fibroblast invasion |
|  | fibroblast spreading |
|  | fibroblast autophagy |
|  | fibroblast transdifferentiation |
|  | fibroblast growth |
|  | fibroblast fate determination |
|  | fibroblast chemotaxis |
|  | fibroblast count |
|  | fibroblast damage |
|  | fibroblast motility |
|  | fibroblast population |
|  | fibroblast size |
|  | fibroblast communication |
|  | fibroblast senescence |
|  | fibroblast proliferative response |
|  | fibroblast death |
|  | fibroblast fate commitment |
|  | fibroblast aggregation |
|  | fibroblast elongation |
|  | fibroblast contact |
|  | fibroblast motion |
|  | fibroblast division |
|  | fibroblast polarity |
|  | fibroblast replicative senescence |
|  | fibroblast transendothelial migration |
|  | fibroblast formation |
|  | fibroblast ratio |
|  | fibroblast interaction |
|  | fibroblast mediated cytotoxicity |
|  | fibroblast regeneration |
|  | fibroblast repertoire |
|  | fibroblast distribution |
|  | fibroblast myofibroblast differentiation |
|  | fibroblast phagocytosis |
|  | fibroblast homing |
|  | fibroblast fate specification |
|  | fibroblast dedifferentiation |
|  | fibroblast destruction |
|  | fibroblast lifespan |
|  | fibroblast structure |
|  | fibroblast cytotoxicity |
|  | fibroblast priming |
|  | fibroblast renewal |
|  | fibroblast morphogenesis |
|  | fibroblast sequestration |
|  | fibroblast anoikis |
|  | fibroblast fate |
|  | fibroblast recognition |
|  | fibroblast homeostasis |
|  | fibroblast stress |
|  | fibroblast volume |
|  | fibroblast anchorage |
|  | fibroblast localization |
|  | fibroblast architecture |
|  | fibroblast swelling |
|  | fibroblast ultrastructure |
|  | fibroblast selection |
|  | fibroblast disruption |

---

|  |  |
| --- | --- |
| Pathway | EGFR -> Expression Targets in Skin |
|  | Elevated Receptors -> Expression Targets in Skin |
|  | NOTCH -> Expression Targets in Skin |
|  | PPARG expressed in cells assotiated with psoriasis |
|  | Neighbors of fibroblast |
|  | 4\_2\_Airway smooth muscle cells proliferation and airway tissue remodeling |
|  | 1\_1\_Dysfunction of coagulation cascade in hemophilia |

---

|  |  |
| --- | --- |
| MedScan ID | 10000151 |

---

|  |  |
| --- | --- |
| Alias | desmocyte |
|  | fibroblast cell |
|  | fibroblast |
|  | fibroblasts |
|  | fibroblastic |
|  | desmocytes |
|  | fibroblast cells |
|  | fibroblastoid cells |
|  | fibroblastoid cell |

---

|  |  |
| --- | --- |
| NCIm ID | C1550637 |

---
